# Protective Effect of Dictyophora Polysaccharides on Sodium Arsenite-Induced Hepatotoxicity: A Proteomics Study

**DOI:** 10.3389/fphar.2021.749035

**Published:** 2021-11-26

**Authors:** Ting Hu, Liming Shen, Qun Huang, Changyan Wu, Huajie Zhang, Qibing Zeng, Guoze Wang, Shaofeng Wei, Shuling Zhang, Jun Zhang, Naseer Ullah Khan, Xiangchun Shen, Peng Luo

**Affiliations:** ^1^ Key Laboratory of Environmental Pollution Monitoring and Disease Control, Ministry of Education, Guizhou Medical University, Guiyang, China; ^2^ School of Public Health, Guizhou Medical University, Guiyang, China; ^3^ Guizhou Provincial Engineering Research Center of Food Nutrition and Health, Guiyang, China; ^4^ Shenzhen Key Laboratory of Marine Biotechnology and Ecology, College of Life Science and Oceanography, Shenzhen University, Shenzhen, China; ^5^ Key Laboratory of Optimal Utilization of Natural Medicine Resources, School of Pharmaceutical Sciences, Guizhou Medical University, Guiyang, China; ^6^ State Key Laboratory of Functions and Applications of Medicinal Plants, Guizhou Medical University, Guiyang, China

**Keywords:** dictyophora polysaccharides, NaAsO_2_, apoptosis, hepatotoxicity, proteomics

## Abstract

The purpose of this study is to understand the mechanism of sodium arsenite (NaAsO_2_)-induced apoptosis of L-02 human hepatic cells, and how Dictyophora polysaccharide (DIP) protects L-02 cells from arsenic-induced apoptosis. The results revealed that DIP pretreatment inhibited NaAsO_2_ induced L-02 cells apoptosis by increasing anti-apoptotic Bcl-2 expression and decreasing pro-apoptotic Bax expression. Proteomic analysis showed that arsenic treatment disrupted the expression of metabolism and apoptosis associated proteins, including ribosomal proteins (RPs). After pretreatment with DIP, the expression levels of these proteins were reversed or restored. For the first time, it was observed that the significant decrease of cytoplasmic RPs and the increase of mitochondrial RPs were related to human normal cell apoptosis induced by arsenic. This is also the first report that the protective effect of DIP on cells was related to RPs. The results highlight the relationship between RPs and apoptosis, as well as the relationship between RPs and DIP attenuating arsenic-induced apoptosis.

## Introduction

Arsenic is known as a serious environmental toxin and human carcinogen (Bhattacharya et al., 2014). According to global epidemiological statistics, more than 200 million people currently suffer from arsenic exposure, particularly in developing countries (Adil et al., 2016). Arsenic exposure caused human health problems involve multiple tissues and organs, especially in the cardiovascular system and liver (Goudarzi et al., 2018). Environmental exposure to arsenic is undoubtedly now a major global public health problem, as well as a serious social and medical problem (Chen et al., 2012; Wang et al., 2020a). Sodium arsenite (NaAsO_2_) is the most toxic substance of different arsenic compounds in our living environment (Duan et al., 2020; Lv et al., 2020). Accumulation of arsenic in the human body can lead to organ damage and tissue canceration. In particular, the liver is one of the target organs of arsenic in humans (Dash et al., 2018; Lv et al., 2020). The cellular and molecular biological experiment has shown that arsenic increases the production of reactive oxygen species (ROS) by inhibiting the activity of antioxidant enzymes. Arsenic can also cause protein oxidation, DNA damage, and apoptosis (Lin et al., 2008; Adil et al., 2015; Goudarzi et al., 2018). However, there is still no broad consensus about the exact mechanism of arsenic-induced toxicity.

Currently, treatments for arsenic poisoning include hemodialysis or chemotherapy, using chelating agents and adsorbents. However, elevated blood pressure and other toxic effects may occur (Saha et al., 2016). Bioactive natural compounds commonly found in dietary plants can treat arsenic-induced toxicity, attracting increased attention to their little or no side effects (Ola-Davies and Akinrinde, 2016; Goudarzi et al., 2018; Perker et al., 2019), and more attention has been paid to their medicinal value (Kanwal et al., 2018; Habtemariam, 2019). Among them, Dictyophora has been reported to act on anti-inflammatory, antioxidant, hypoglycemic, and lipid-lowering effects (Zhang et al., 2016; Han et al., 2017; Wang Y. et al., 2019). Furthermore, the polysaccharide extracted from Dictyophora has a protective effect on the liver (Wang et al., 2019a; Wang et al., 2019b; Hu et al., 2020). However, the effect of Dictyophora polysaccharide (DIP) on sodium arsenite induced hepatotoxicity is still unclear.

In this study, DIP was shown to protect human normal liver cells L-02 from the sodium arsenic induced toxicity, and a comparative proteomics analysis based on iTRAQ (isobaric tags for relative and absolute quantification) was performed to explore the molecular mechanism of arsenic-induced apoptosis and the protective effect of DIP on arsenic-induced hepatotoxicity.

## Materials and Methods

### Chemicals

Food-grade Dictyophora was provided by Zhijin Sifang Hongye (Zhijin City, Guizhou Province, China). Sodium arsenite (NaAsO_2_) was obtained from Sigma Chemical Corp (St. Louis, MO, United States). Human normal hepatocytes (L-02 cells) were purchased from the Shanghai Cell Bank of the Chinese Academy of Sciences (Shanghai, China). RPMI 1640 cell culture medium, trypsin, and fetal bovine serum (FBS) were purchased from Gibco Company (California, United States). Phosphate buffer saline (PBS) was purchased from Zhongsha Jinqiao Biotechnology Co., Ltd. (Beijing, China). Dimethyl sulfoxide (DMSO) was obtained from Sigma (St. Louis, MO, United States). The BCA protein detection kit, protein sample buffer, and Western blot analysis gel preparation kit were purchased from Beyotime Biotechnology Co., Ltd. (Shanghai, China). Protein molecular weight markers were obtained from Fermentas (Burlington, Canada). Polyvinylidene fluoride (PVDF) film and enhanced chemiluminescence (ECL) kit were purchased from Bio-Rad (California, United States). Cell Counting Kit-8 (CCK-8), RIPA lysis buffer, rabbit anti-human Bcl-2, Bax, β-actin, GAPDH, ribosomal protein S5 (Rps5), and 14-3-3 protein sigma (SFN) antibodies and horseradish peroxidase (HRP) labeled secondary antibodies were purchased from Boster Biological Technology, Ltd. (Boster, Wuhan, China).

### Dictyophora Polysaccharide Preparation

The separation and purification of DIP was performed as previously described with minor modifications (Liao et al., 2015; Zhang et al., 2016; Yu et al., 2017; Hu et al., 2020). Briefly, the fruit body of Dictyophora was dried at 45°C for 2 h, ground into powder, extracted in boiling water for 3 h at a water-to-material ratio (1:20), and then centrifuged at 4,000 × g for 15 min. The supernatant was extracted and concentrated at 50°C using a rotary evaporator (R-215, Buchi, Switzerland). The concentrated extract was collected and proteins were removed using the Sevage method. The crude polysaccharides of Dictyophora were obtained by freeze-drying after overnight precipitation with 4 volumes of 100% ethanol. The content of sugar was determined by using phenol-sulfuric acid colorimetric assay. The content was 84.13%. According to our experimental data, 10 mg DIP can be obtained from 80 mg of dried Dictyophora powder.

### Preparation of Dictyophora Polysaccharide Stock Solution

The dry product of 400 mg DIP was dissolved in 20 ml serum-free DMEM medium to prepare a final concentration of 20 mg/ml mother liquor, filtered and stored at −20°C. When in use, it is diluted to the corresponding concentration with serum-free DMEM medium.

### Cell Culture

L-02 cells were cultured in a 5% CO_2_ incubator at 37°C. The control group used DMEM high glucose medium containing 10% FBS and 1% penicillin/streptomycin, and the treatment group was pretreated with NaAsO_2_ or DIP for 4 h and then exposed to NaAsO_2_. All experiments were performed 24 h after cell inoculation.

### Cell Counting Kit-8 Assay

The cell viability of L-02 cells was measured by CCK-8 assay. Cells were seeded in 96-well plates at a density of 1 × 10^4^ cells per well, and were treated with different concentration of NaAsO_2_ for 24 h. In order to explore the intervention effect of DIP, L-02 cells were pretreated with different concentration DIP solution for 4 h, and then treated with of 10 μM NaAsO_2_ for 24 h. In addition, the viability of cells pretreated with DIP (80 μg/ml) and then exposed to different concentrations sodium arsenate was also investigated. CCK-8 reagent was added to each well and incubated at 37°C for 2 h according to the manufacturer protocol. Microplate Reader (Thermo Fisher Scientific) was used to measure the absorbance at 450 nm to determine cell viability.

### Annexin V/PI Assay

The percentage of apoptotic cells was detected by Annexin V-FITC apoptosis detection kit (Beyotime, Shanghai, China). After NaAsO_2_ (10 μM) treatment or DIP (80 μg/ml) pretreatment followed by exposure to NaAsO_2_, the L-02 cells were collected and washed with PBS, and re-suspended by adding 100 μL binding buffer, and then incubated with 5 µL Annexin V-FITC, and 10 µL PI at room temperature. The apoptosis rate was detected by flow cytometry (ACEA NovoCyte, United States).

### Protein Extraction for Proteomics Analysis

After treatment with NaAsO_2_ (10 μM) or DIP (80 μg/ml) pretreatment and then exposed to 10 μM NaAsO_2_, L-02 cells were rinsed twice with ice-cold PBS. The cells were harvested and resuspended in lysis buffer (8 M urea, 2 mM EDTA, 10 mM DTT, and 1% protease inhibitor cocktail). After sonicated and centrifuged at 13,000 g at 4°C for 10 min to remove debris, the protein in supernatant was precipitated with cold acetone for 2 h at −20°C. After centrifugation at 4°C at 12,000 × g for 10 min, the protein deposit was redissolved by urea buffer [8 M urea, 100 mM TEAB (triethylammonium bicarbonate)]. The protein concentration was detected using Bradford protein assay kit (Beyotime).

### Trypsin Digestion

For trypsin digestion, 100 μg protein of each sample was first reduced with 10 mM DTT at 37°C for 60 min and then alkylated with 55 mM iodoacetamide (IAM) at room temperature for 30 min in darkness. The urea content of protein extract was diluted by adding 100 mM TEAB less than 2 M. The protein pool of each sample was digested with trypsin with the ratio of protein: trypsin = 50:1 mass ratio at 37°C overnight and 100:1 for a second digestion at 4 h.

### Peptides Isobaric Tags for Relative and Absolute Quantification Labeling

After trypsin digestion, the peptides were desalted by Strata X SPE column and vacuum-dried. The digested peptides were then labeled with the iTRAQ reagents (AB Sciex), as follows: the control group was labeled with iTRAQ 113 and 114, while NaAsO_2_ treatment group with iTRAQ 115 and 116, and DIP pretreatment and NaAsO_2_ treatment group with iTRAQ 117 and 118. Briefly, peptides were reconstituted in 20 μl 500 mM TEAB and processed according to the manufacturer’s protocol for 8-plex iTRAQ kit (AB Sciex, Foster City, CA, United States). One unit of iTRAQ reagent was applied to the peptide solution after thawed and dissolved in 50 μL isopropanol. The peptide mixtures were incubated for 2 h at room temperature, then pooled and dried by vacuum centrifugation.

### High-Performance Liquid Chromatographic Fractionation

The dried and labeled peptide was reconstituted with HPLC solution A [2% ACN (acetonitrile), pH 10] and then fractionated into high pH reverse-phase HPLC fractions using Waters Bridge Peptide BEH C18 (130 Å, 3.5 μm, 4.6 × 250 mm). Peptides were first separated by a gradient of 2–98% acetonitrile in pH 10 at a speed of 0.6 ml/min over 88 min into 48 fractions. The peptides were then mixed into 15 fractions and dried by vacuum centrifugation. The peptide fractions were desalted using Ziptip C18 (Millipore, MA, United States). Samples were finally dried under vacuum and kept at −20°C until they were analyzed by MS (mass spectrometry).

### High-Resolution LC-MS/MS Analysis

The experiment was then performed by NanoLC 1000 LC-MS/MS using a Proxeon EAsY-nLC 1000 coupled to Q-Exactive mass spectrometer (Thermo Fisher Scientific, United States). Trypsin digestion fractions were reconstituted in 0.1% FA (formic acid) and immediately charged to the reversed-phase pre-column (Acclaim PepMap®100 C18, 3 μm, 100 Å, 75 μm × 2 cm) at 5 μl/min in 100% solvent A (0.1 M acetic acid in water). Next, peptides eluted from the trap column were loaded into a reversed-phase analytical column (Acclaim PepMap® RSLC C18, 2 μm, 100 Å, 50 μm × 15 cm). The gradient was comprised of an increase from 0 to 8% solvent B 0.1% FA in 98% ACN over 5 min, 8–25% solvent B over 35 min, 25–98% solvent B during 10 min and keep in 98% in 8 min at a constant flow rate of 300 nL/min at EAsY-nLC 1000 system. The eluent was sprayed from an NSI source at an electrospray voltage of 2.5 kV and then analyzed by tandem mass spectrometry (MS/MS) in Q Exactive. The mass spectrometer was operated in data-dependent mode, automatically switching between MS and MS/MS. Full-scan MS spectra (from m/z 300–2000) were acquired in the Orbitrap with a resolution of 70,000. Ion fragments were detected in the Orbitrap at a resolution of 17,500. The 15 most intense precursors were selected for subsequent decision tree-based ion trap HCD fragmentation at the collision energy of 32% in the MS survey scan with 10.0 s dynamic exclusion.

### Data Processing and Isobaric Tags for Relative and Absolute Quantification

The resulting MS/MS raw data was searched against the transcriptome database using Sequest software integration in Proteome Discoverer (version 1.3, Thermo Scientific). The quest parameters were as follows: trypsin as a digestion enzyme, two missing cleavages, oxidized methionine, acetylation in N-Term, iTRAQ modification at the N-terminus of the peptide and iTRAQ 8-plex (K, Y) as the variable modification, fixed modifications like carbamidomethyl (C). The peptide mass tolerance and fragment mass tolerance were set to 20 ppm and 0.05 Da, respectively. A decoy database search strategy was adopted to estimate the false discovery rate (FDR) for peptide identification. For this study, a high peptide confidence (1% FDR) was selected. The cut-off values of 1.2-fold for up-regulated and 0.83-fold for down-regulated proteins, *p*-value < 0.05, were set as differentially expressed proteins (DEPs) (Shen et al., 2021).

### Bioinformatics Analysis

Bioinformatics analysis was performed by using OMICSBEAN online tools (http://www.omicsbean.cn/) and String (Search Tool for the Retrieval of Interacting Genes/Proteins, version 9.1, http://string-db.org/) database. DEPs were analyzed by GO (gene ontology) annotation, KEGG (Kyoto Encyclopedia of Genes and Genomes) pathways, and protein-protein interaction (PPI) networks (Iqbal et al., 2018; Wei et al., 2018). Functional interaction network analysis was conducted using the ClueGO Cytoscape plugin (Bindea et al., 2009). The GO categories and pathways searched include biological processes (BP), cellular components (CC), molecular function (MF), KEGG (Kyoto Encyclopedia of Genes and Genomes), REACTOME, and Wiki pathway.

### Western Blot Analysis

L-02 cells were incubated in 6-well plates. After being treated with NaAsO_2_ or DIP as indicated, cells were collected and lysed with RIPA buffer containing 1 mM PMSF and 1% protease inhibitor cocktail. The protein concentration was measured using the BCA kit. After SDS-PAGE electrophoresis transformation, the separated proteins were transferred to a polyvinylidene fluoride (PVDF) membrane. The membrane was blocked with 5% skimmed milk at room temperature for 2 h and incubated with the indicated primary antibodies overnight at 4°C. After incubated with HRP-conjugated second antibody at room temperature for 1 h, the blotting signal was generated by chemiluminescence using an enhanced chemiluminescence (ECL) kit (Thermo Fisher Scientific). The gray value of protein band was analyzed by Image Labe software, and the ratio of target protein to internal reference gray value was used to reflect the expression of protein.

### Statistical Analysis

Statistical analysis was carried out with SPSS Version 20.0 (SPSS Software, Chicago, IL, United States). The experimental data is shown as the means ± SD. Single factor analysis of variance (ANOVA) was used to detect the different distribution of various groups. The distribution of biometric values is normalized by logarithmic transformation. The statistically significant level was *p* < 0.05.

## Results

### Detection of Components of DIP by High-Performance Liquid Chromatographic

**FIGURE 1:**
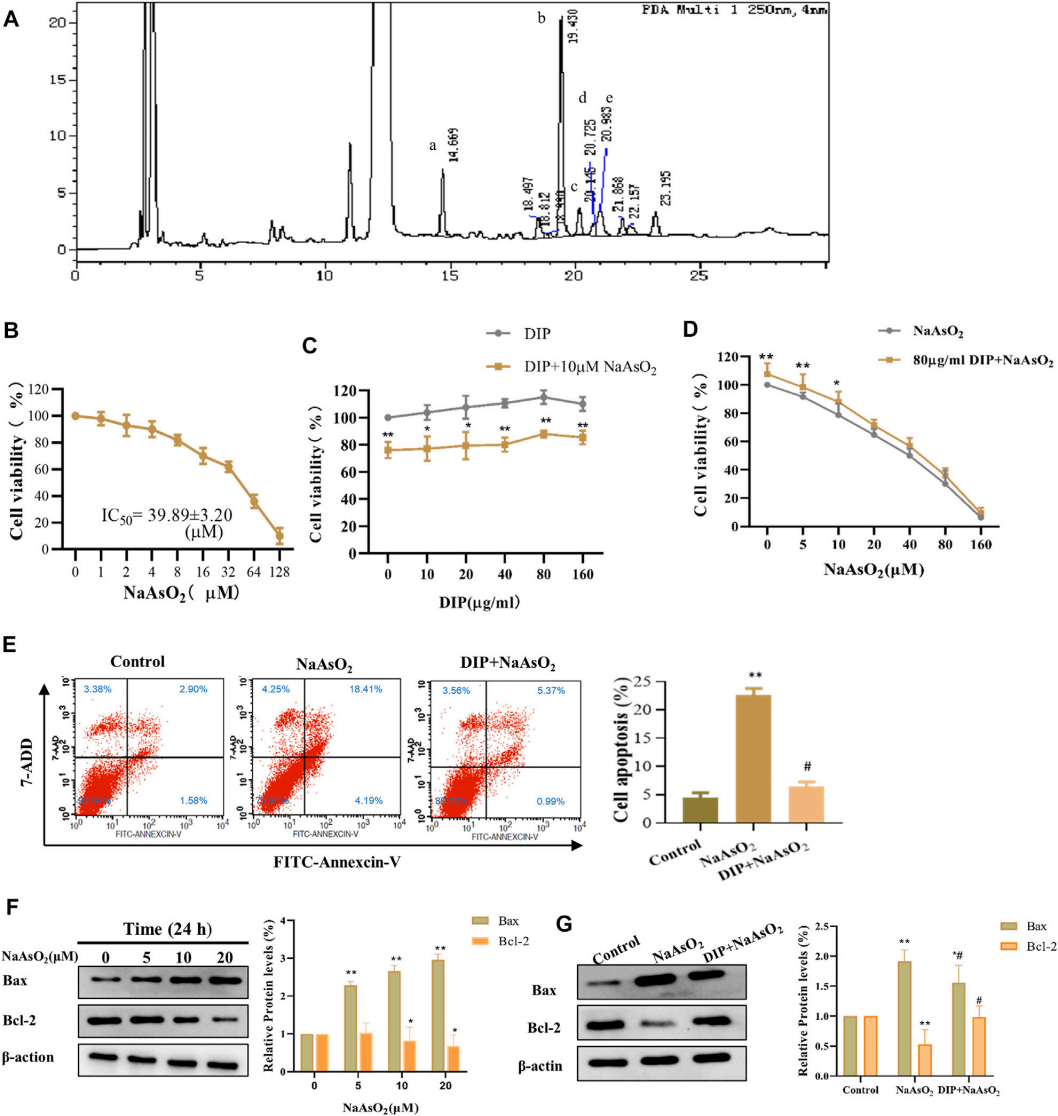
DIP against NaAsO_2_-induced hepatotoxicity in L-02 cells. **(A)** HPLC profile of sporocarp polysaccharide of Dictyophora. (a) D-mannose. (b) D-glucose. (c) D-galactose. (d) D-xylose. (e) L-fucose. **(B)** The viability of L-02 cells treated with different concentrations of NaAsO_2_ (CCK-8 assay). **(C)** The viability of L-02 cells exposed to arsenic (10 μM NaAsO_2_) with or without pretreatment with different concentrations of DIP. **(D)** The viability of L-02 cells pretreated with DIP (80 μg/ml) and then exposed to different concentrations of NaAsO_2_. **(E)** Flow cytometry profiles showing the distribution of cells **(Left)**. Plot showing the proportion of apoptotic cells **(Right)**. Compared with control group, **p < 0.01; compared with NaAsO_2_ group, #*p* < 0.05. **(F)** Western blot analysis of Bax or Bcl-2 protein expression levels when treated with different arsenic concentrations **(Left)**. Plot showing Bax or Bcl-2 protein expression levels **(Right)**. (**G**) Western blot analysis of Bax or Bcl-2 protein expression levels when pretreated with DIP and then treated with arsenic **(Left)**. Plot showing Bax or Bcl-2 protein expression levels **(Right)**. Compared with the control group, *p < 0.05, **p < 0.01. Compared with the control group, *p < 0.05, **p < 0.01; compared with NaAsO_2_ group, #*p* < 0.05.

The HPLC detection showed that the monosaccharides in the Dictyophora polysaccharides (DIP) were D-mannose, D-glucose, D-galactose, D-xylose, L-fucose (Figure 1A), and their contents were 15.27, 523.57, 27.80, 10.01, and 17.84 mg/L respectively, of which D-glucose accounted for 88.07% (Supplementary Table S1).

### DIP Antagonizes the Suppression Effect of NaAsO_2_ on L-02 Cell Viability

To investigate the cytotoxicity of sodium arsenite to L-02 cells, L-02 cells were treated with NaAsO_2_ treatment. We found that NaAsO_2_ reduced the viability of L-02 cells in a dose-dependent manner. The IC_50_ value was 39.89 ± 3.20 μM (Figure 1B). However, DIP pretreatment significantly improved L-02 cells viability, even when exposed to 1/8 of IC_50_ (5 μM) and 1/4 of IC_50_ (10 μM) NaAsO_2_. Pretreatment with 80 μg/ml DIP enabled L-02 cells to tolerate 10 μM sodium arsenite (Figures 1C,D). The results indicated that DIP has the potential to antagonize sodium arsenite cytotoxicity.

### DIP Inhibits NaAsO_2_-Induced Apoptosis in L-02 Cells

Considering sodium arsenite is recognized as an apoptosis inducer (Bashir et al., 2006; Chen et al., 2012; Sun et al., 2017), we then investigated whether NaAsO_2_ could induce apoptosis in L-02 cells. Flow cytometry analysis showed that the proportion of apoptotic cells increased significantly after treatment with 10 μM NaAsO_2_ (Figure 1E). Furthermore, with the increasing dosage of NaAsO_2_ treatment, pro-apoptotic protein Bax was up-regulated, while anti-apoptotic Bcl-2 protein was down-regulated in L-02 cells (Figure 1F), suggesting NaAsO_2_ can induce the apoptosis of L-02 cells. However, DIP pretreatment not only significantly reduced the apoptosis induced by NaAsO_2_ (Figure 1E) but also reversed the expression of Bax and Bcl-2 (Figure 1G), revealing the protective role of DIP in resisting sodium arsenite-induced apoptosis of L-02 cells.

### Overview of Protein Expression Characteristics of Different Groups

**FIGURE 2:**
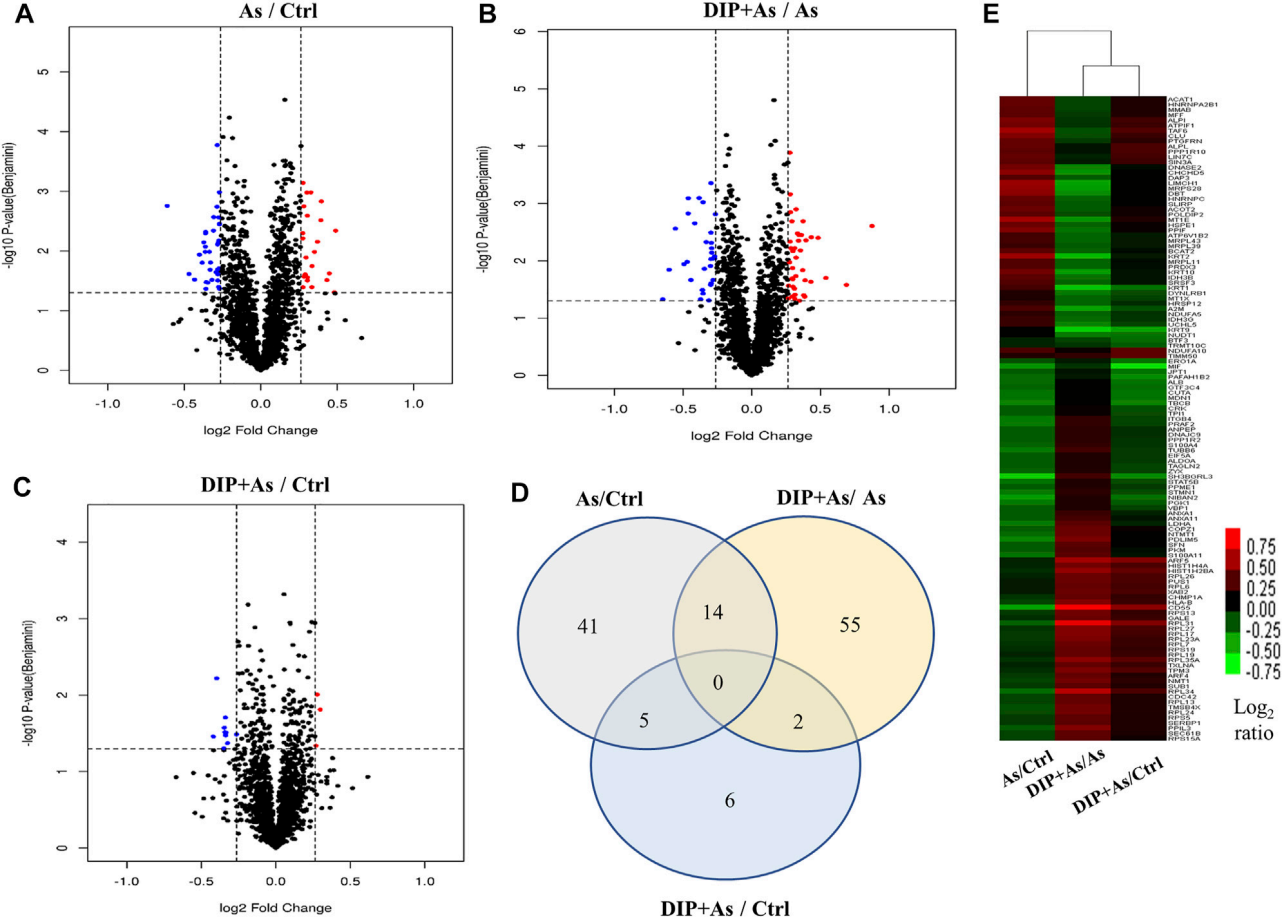
The differentially expressed proteins identified in different groups. **(A)** Volcano plots depicted the distribution of proteins in As/Ctrl group. **(B)** Volcano plots depicted the distribution of proteins in DIP + As/As group. **(C)** Volcano plots depicted the distribution of proteins in DIP + As/Ctrl group. **(D)** Venn diagrams of DEPs between the As/Ctrl group, DIP + As/As group, and DIP + As/Ctrl group. **(E)** Cluster map comparing the DEPs identified in As/Ctrl, DIP + As/As, and DIP + As/Ctrl groups. Red color indicates higher expression, green indicates lower expression, and black indicates similar expression levels. **(A–C)**: The log_2_ fold change (FC) is plotted versus the –log_10_ of the *p*-value (Benjamini). Red dots = hits with *p* < 0.05 and mean log_2_FC > 0.83; blue dots = hits with *p* < 0.05 and means |log_2_FC| < 0.83.

The iTRAQ analysis was further performed to explore the molecular mechanism of DIP against sodium arsenite-induced cytotoxicity in L-02 cells. We identified the protein expression profile in L-02 cells treated with 10 μM NaAsO_2_ compared with the control group (As/Ctrl group). After DIP pretreatment, the protein expression characteristics of arsenic treated group were also analyzed (DIP + As/As group). A total of 2,876 proteins were identified. Among them, 60, 71, and 13 proteins were identified as DEPs in As/Ctrl group, DIP + As/As group, and DIP + As/Ctrl groups (Figures 2A–C; Table 1), respectively. Of these, 14 DEPs were found to be common between the As/Ctrl group and DIP + As/As group, with the opposite expression trend in these two groups (named as reversed proteins; Figure 2D and Table 1). Cluster analysis showed that the protein expression characteristics of DIP + As/As group and DIP + As/Ctrl were more similar, but almost opposite to As/Ctrl group (Figure 2E).

**TABLE 1:** The differentially expressed proteins identified in different groups in this study.

Protein Name^a^	Gene name	Uniprot No.	As/Ctrl ^b^	DIP+As/As ^b^	DIP+As/ Ctrl ^b^
**10 kDa heat shock protein, mitochondrial**	HSPE1	P61604	**1.25**	**0.82**	1.02
**14-3-3 protein sigma**	SFN	P31947	**0.83**	**1.21**	1.00
28S ribosomal protein S29, mitochondrial	DAP3	P51398	1.20	**0.83**	0.99
2-iminobutanoate/2-iminopropanoate deaminase	HRSP12	P52758	1.12	**0.82**	0.91
40S ribosomal protein S19	RPS19	P39019	0.91	**1.23**	1.12
60S ribosomal protein L17	RPL17	P18621	0.88	**1.32**	1.15
60S ribosomal protein L23a	RPL23A	P62750	0.89	**1.29**	1.14
60S ribosomal protein L27	RPL27	P61353	0.89	**1.32**	1.17
60S ribosomal protein L34	RPL34	P49207	0.80	**1.47**	1.17
60S ribosomal protein L35a	RPL35A	P18077	0.89	**1.35**	1.20
60S ribosomal protein L6	RPL6	Q02878	0.95	**1.23**	1.16
60S ribosomal protein L7	RPL7	P18124	0.90	**1.25**	1.13
7,8-dihydro-8-oxoguanine triphosphatase	NUDT1	P36639	1.02	**0.78**	**0.79**
Acetyl-CoA acetyltransferase, mitochondrial	ACAT1	P24752	**1.23**	0.86	1.06
Activated RNA polymerase II transcriptional coactivator p15	SUB1	P53999	0.89	**1.21**	1.07
**Acyl-coenzyme A thioesterase 2, mitochondrial**	ACOT2	P49753	**1.26**	**0.82**	1.04
Adapter molecule crk	CRK	P46108	**0.83**	1.02	0.84
ADP-ribosylation factor 4	ARF4	P18085	0.88	**1.23**	1.08
ADP-ribosylation factor 5	ARF5	P84085	0.92	**1.40**	1.30
Alkaline phosphatase, intestinal	ALPI	P09923	**1.29**	0.90	1.16
Alkaline phosphatase, tissue-nonspecific isozyme	ALPL	P05186	**1.23**	0.96	1.18
Alpha-2-macroglobulin	A2M	P01023	1.17	**0.72**	0.84
Alpha-taxilin	TXLNA	P40222	0.92	**1.23**	1.13
Aminopeptidase N	ANPEP	P15144	**0.82**	1.10	0.91
Annexin A1	ANXA1	P04083	**0.82**	1.14	0.93
Annexin A11	ANXA11	P50995	**0.83**	1.15	0.95
ATPase inhibitor, mitochondrial	ATPIF1	Q9UII2	**1.26**	0.90	1.13
Branched-chain-amino-acid aminotransferase, mitochondrial	BCAT2	O15382	1.20	**0.81**	0.97
Cell division control protein 42 homolog	CDC42	P60953	0.87	**1.22**	1.06
Charged multivesicular body protein 1a	CHMP1A	Q9HD42	0.90	**1.23**	1.11
Clusterin	CLU	P10909	**1.31**	0.86	1.12
**Coatomer subunit zeta-1**	COPZ1	P61923	**0.83**	**1.24**	1.03
Cob(I)yrinic acid a,c-diamide adenosyltransferase, mitochondrial	MMAB	Q96EY8	**1.22**	0.87	1.06
Complement decay-accelerating factor	CD55	P08174	0.71	**1.85**	1.33
**Deoxyribonuclease-2-alpha**	DNASE2	O00115	**1.27**	**0.77**	0.98
Dihydrolipoamide branched chain transacylase E2	DBT	P11182	1.33	**0.75**	1.01
DNA polymerase delta interacting protein 2	POLDIP2	Q9Y2S7	**1.22**	0.85	1.04
DnaJ homolog subfamily C member 9	DNAJC9	Q8WXX5	**0.83**	1.10	0.91
Dynein light chain roadblock-type 1	DYNLRB1	Q9NP97	1.08	**0.78**	0.85
ERO1-like protein alpha	ERO1A	Q96HE7	0.84	0.94	**0.79**
Eukaryotic translation initiation factor 5A-1	EIF5A	P63241	**0.82**	1.07	0.88
Fructose-bisphosphate aldolase A	ALDOA	P04075	**0.83**	1.06	0.88
General transcription factor 3C polypeptide 4	GTF3C4	Q9UKN8	**0.82**	0.99	0.81
Glycylpeptide N-tetradecanoyltransferase 1	NMT1	P30419	0.87	**1.26**	1.09
Heterogeneous nuclear ribonucleoproteins A2/B1	HNRNPA2B1	P22626	**1.23**	0.86	1.06
**Heterogeneous nuclear ribonucleoproteins C1/C2**	HNRNPC	P07910	**1.23**	**0.81**	1.01
Histone cluster 1 H4 family member f	HIST1H4A	P62805	0.92	**1.27**	1.17
Histone H2B type 1-A	HIST1H2BA	Q96A08	0.91	**1.35**	**1.23**
HLA class I histocompatibility antigen, B-7 alpha chain	HLA-B	P30479	0.87	**1.31**	1.14
Integrin beta-4	ITGB4	P16144	**0.78**	1.12	0.87
**Isocitrate dehydrogenase (NAD) subunit beta, mitochondrial**	IDH3B	O43837	**1.21**	**0.79**	0.95
Isocitrate dehydrogenase (NAD) subunit gamma, mitochondrial	IDH3G	P51553	1.13	**0.79**	0.90
Jupiter microtubule associated homolog 1	JPT1	Q9UK76	0.83	0.96	**0.79**
Keratin, type I cytoskeletal 10	KRT10	P13645	1.28	**0.72**	0.95
Keratin, type I cytoskeletal 9	KRT9	P35527	1.02	**0.65**	0.71
Keratin, type II cytoskeletal 1	KRT1	P04264	1.16	**0.67**	0.79
**Keratin, type II cytoskeletal 2 epidermal**	KRT2	P35908	**1.38**	**0.68**	0.95
Lactate dehydrogenase A	LDHA	P00338	**0.78**	1.20	0.93
LIM and calponin homology domains 1	LIMCH1	Q9UPQ0	1.39	0.71	1.00
Macrophage migration inhibitory factor	MIF	P14174	0.74	0.89	0.63
Metallothionein-1E	MT1E	P04732	1.41	**0.74**	1.08
Metallothionein-1X	MT1X	P80297	1.06	**0.81**	0.87
Midasin	MDN1	Q9NU22	**0.78**	1.00	0.78
Mitochondrial coiled-coil-helix-coiled-coil-helix domain containing proteins	CHCHD5	Q9BSY4	**1.34**	0.74	0.98
Mitochondrial fission factor	MFF	Q9GZY8	**1.21**	0.89	1.07
Mitochondrial import inner membrane translocase subunit TIM50	TIMM50	Q3ZCQ8	1.07	1.12	**1.21**
Mitochondrial ribosomal protein L11	MRPL11	Q9Y3B7	1.19	**0.82**	0.97
Mitochondrial ribosomal protein L39	MRPL39	Q9NYK5	1.14	**0.83**	0.95
Mitochondrial ribosomal protein L43	MRPL43	Q8N983	1.15	**0.82**	0.94
**Mitochondrial ribosomal protein S28**	MRPS28	Q9Y2Q9	**1.35**	**0.73**	0.99
NADH dehydrogenase (ubiquinone) 1 alpha subcomplex subunit 5	NDUFA5	Q16718	1.10	**0.82**	0.90
NADH dehydrogenase (ubiquinone) 1 alpha subcomplex subunit 10, mitochondrial	NDUFA10	O95299	1.17	1.03	**1.21**
Niban-like protein 1	NIBAN2	Q96TA1	**0.72**	1.08	**0.79**
N-terminal Xaa-Pro-Lys N-methyltransferase 1	NTMT1	Q9BV86	0.80	**1.24**	1.00
Paired amphipathic helix protein Sin3a	SIN3A	Q96ST3	**1.21**	0.94	1.14
**PDZ and LIM domain protein 5**	PDLIM5	Q96HC4	**0.76**	**1.30**	0.99
**Peptidyl-prolyl cis-trans isomerase**	PPIL3	Q9H2H8	**0.82**	**1.31**	1.07
**Peptidyl-prolyl cis-trans isomerase F, mitochondrial**	PPIF	P30405	**1.31**	**0.78**	1.03
Phosphoglycerate kinase 1	PGK1	P00558	**0.78**	1.07	0.83
Plasminogen activator inhibitor 1 RNA-binding protein	SERBP1	Q8NC51	0.88	**1.21**	1.06
Platelet-activating factor acetylhydrolase IB subunit beta	PAFAH1B2	P68402	**0.79**	0.95	0.75
PRA1 family protein 2	PRAF2	O60831	**0.78**	1.11	0.86
Prefoldin subunit 3	VBP1	P61758	**0.83**	1.05	0.87
Pre-mRNA-splicing factor SYF1	XAB2	Q9HCS7	0.94	**1.25**	1.18
Prostaglandin F2 receptor negative regulator	PTGFRN	Q9P2B2	1.23	0.89	1.09
Protein CutA	CUTA	O60888	**0.78**	1.01	**0.79**
Protein lin-7 homolog C	LIN7C	Q9NUP9	**1.22**	0.93	1.14
Protein phosphatase inhibitor 2	PPP1R2	P41236	**0.82**	1.10	0.90
Protein phosphatase methylesterase 1	PPME1	Q9Y570	**0.83**	1.07	0.89
**Protein S100-A11**	S100A11	P31949	**0.79**	**1.22**	0.96
Protein transport protein Sec61 subunit beta	SEC61B	P60468	0.87	**1.21**	1.05
Pyruvate kinase PKM	PKM	P14618	**0.82**	1.19	0.98
Ribosomal protein L13	RPL13	P26373	0.87	**1.23**	1.07
Ribosomal protein L19	RPL19	P84098	0.93	**1.23**	1.14
Ribosomal protein L24	RPL24	P83731	0.85	**1.28**	1.09
Ribosomal protein L26	RPL26	P61254	0.93	**1.25**	1.16
Ribosomal protein L31	RPL31	P62899	0.82	**1.62**	1.31
Ribosomal protein S13	RPS13	P62277	0.88	1.27	1.11
Ribosomal protein S15a	RPS15A	P62244	0.86	1.21	1.05
Ribosomal protein S5	RPS5	P46782	0.88	1.21	1.07
S100 calcium binding protein A4	S100A4	P26447	**0.81**	1.12	0.90
Serine/arginine-rich splicing factor 3	SRSF3	P84103	1.18	**0.81**	0.96
Serine/threonine-protein phosphatase 1 regulatory subunit 10	PPP1R10	Q96QC0	**1.22**	0.96	1.18
Serum albumin	ALB	P02768	**0.80**	0.98	**0.79**
SH3 domain-binding glutamic acid-rich-like protein 3	SH3BGRL3	Q9H299	**0.65**	1.15	**0.75**
Signal transducer and activator of transcription 5B	STAT5B	P51692	**0.79**	1.08	0.86
**SRA stem-loop-interacting RNA-binding protein, mitochondrial**	SLIRP	Q9GZT3	**1.21**	**0.83**	1.01
Stathmin	STMN1	P16949	**0.78**	1.09	0.85
Thioredoxin-dependent peroxide reductase, mitochondrial	PRDX3	P30048	1.20	**0.81**	0.97
Thymosin beta-4	TMSB4X	P62328	0.85	**1.27**	1.08
Transcription factor BTF3	BTF3	P20290	0.92	0.87	**0.80**
Transcription initiation factor TFIID subunit 6	TAF6	P49848	**1.40**	0.84	1.18
Transgelin-2	TAGLN2	P37802	**0.82**	1.06	0.87
Triosephosphate isomerase 1	TPI1	P60174	**0.83**	1.03	0.85
tRNA methyltransferase 10 homolog C	TRMT10C	Q7L0Y3	0.93	0.89	**0.83**
tRNA pseudouridine synthase A	PUS1	Q9Y606	0.94	**1.24**	1.16
Tropomyosin alpha-3 chain	TPM3	P06753	0.91	**1.29**	1.18
Tubulin beta-6 chain	TUBB6	Q9BUF5	**0.77**	1.15	0.89
Tubulin-folding cofactor B	TBCB	Q99426	**0.76**	1.00	**0.76**
Ubiquitin carboxyl-terminal hydrolase isozyme L5	UCHL5	Q9Y5K5	1.13	**0.77**	0.87
UDP-glucose 4-epimerase	GALE	Q14376	0.91	**1.25**	1.13
V-type proton ATPase subunit B, brain isoform	ATP6V1B2	P21281	1.16	**0.81**	0.94
Zyxin	ZYX	Q15942	**0.81**	1.07	0.86

a Bold: the reversed proteins identified in the present study.

b Fold change, bold, p < 0.05.

### Protein Expression Profile of NaAsO_2_-Treated L-02 Cells Compared With Natural Growth L-02 Cells (As/Ctrl Group).

**FIGURE 3:**
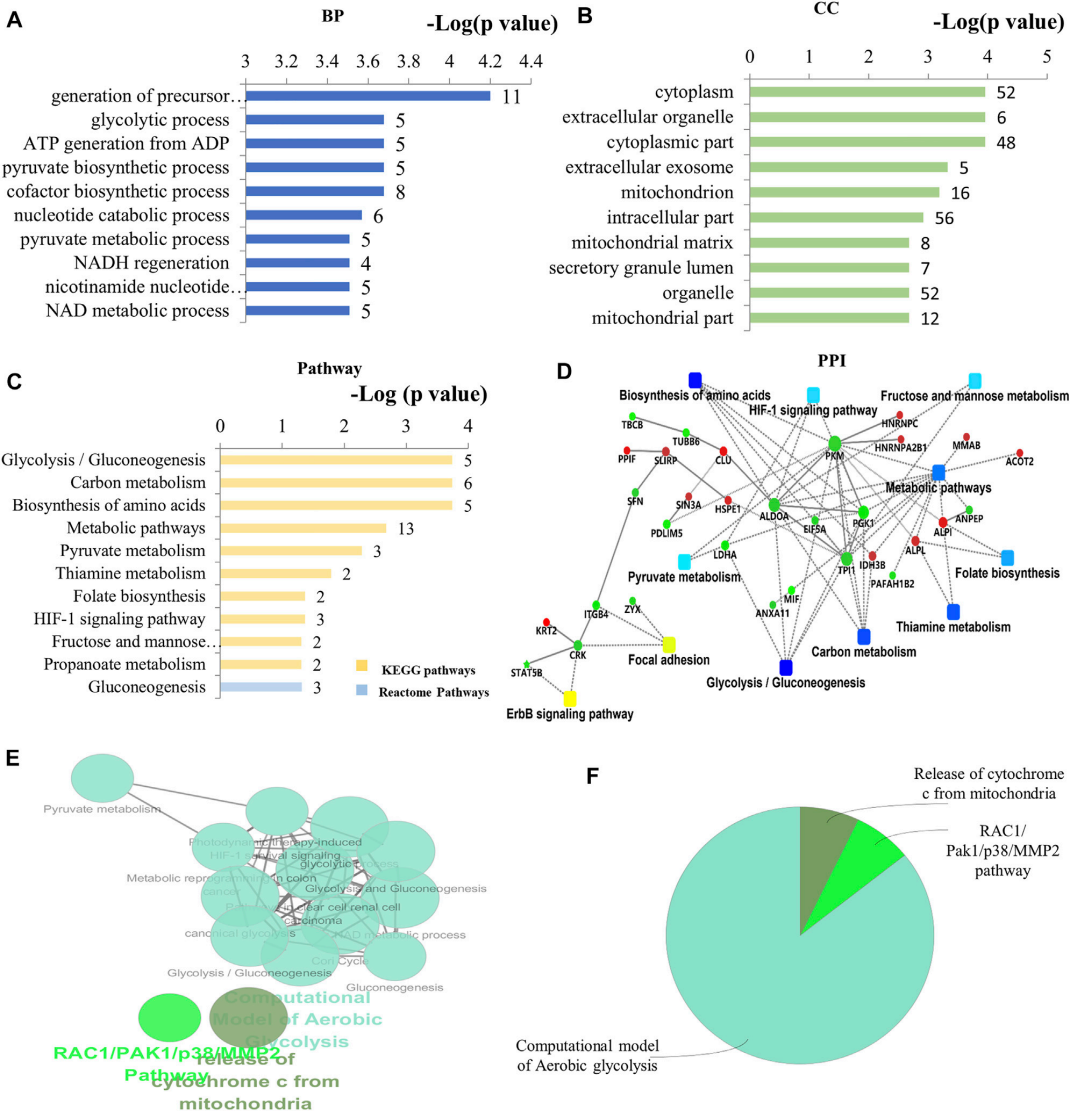
Bioinformatics analysis of the differentially expressed proteins in As/Ctrl group. **(A)** The top 10 ranking of BP associated with differentially expressed proteins. **(B)** The top 10 ranking of CC associated with differentially expressed proteins. **(C)** The significantly enriched pathways linked to differentially expressed proteins. **(D)** PPI network linked to the differentially expressed proteins in As/Ctrl group. Red: the expression of these proteins was up-regulated. Green: the expression of these proteins was down-regulated. **(E)** Functional interaction network analysis was performed by using ClueGO cytoscape plugin. **(F)** Overview the specific cluster. **(A–C)** Number of proteins associated with each category for gene-enrichment analysis is shown on the right of each term bar.

By bioinformatics analysis, the BP, CC, MF, and KEGG pathways associated with these 60 DEPs in As/Ctrl group are presented in Figures 3A–D and Supplementary Table S2–5. The BP related to DEPs in As/Ctrl group involved mainly in the generation of precursor metabolites and energy, glycolytic process, ATP generation from ADP, pyruvate biosynthesis process, and NADH regeneration, etc. (Figure 3A)**.** In particular**,** 14 DEPs were related to regulation of apoptotic process including ALB, ANXA1, ATPIF1, CLU, FAM129B, HSPE1, LDHA, MFF, MIF, PPIF, PPP1R10, SFN, SIN3A, and STAT5B (Supplemenntary Table S2). Four DEPs (CLU, MFF, PPIF, and SFN) were associated with apoptotic mitochondrial changes, 3 DEPs (MFF, POLDIP2, and SLIRP) were related to mitochondrion morphogenesis, and 3 DEPs (CLU, MFF, and SFN) were involved in the release of cytochrome c from mitochondria (Supplementary Table S2). The DEPs were mainly distributed in the cytoplasm, cytoplasmic parts, mitochondrion, and mitochondrial matrix, etc. (Figure 3B; Supplementary Table S3). The MF of these DEPs included protein binding, calcium-dependent protein binding, alkaline phosphatase activity, and protein phosphatase inhibitor activity, etc. (Supplementary Table S4).

After NaAsO_2_ treatment, KEGG pathway analysis showed that most DEPs were involved in metabolic pathways, including glycolysis, carbon metabolism, pyruvate metabolism, amino acid biosynthesis and metabolic thiamine, folic acid biosynthesis, etc. (Figure 3C). Seven (7) DEPs were involved in these metabolism-related pathways, including ACAT1, ALDOA, IDH3B, LDHA, PGK1, PKM, and TPI1. Among them, 5 DEPs were down-regulated in L-02 cells, including ALDOA, LDHA, PGK1, PKM, TPI1 (Table 1; Supplementary Table S5). Other signal transduction pathways included the HIF-1 signaling pathway. The DEPs associated with this pathway include ALDOA, LDHA, and PGK1, which have also been down-regulated (Table 1; Supplementary Table S5). The PPI network associated with the DEPs is shown in Figure 3D. Consistently, these DEPs were enriched into metabolism-related pathways, including glycolysis/gluconeogenesis, glycosylcarbon metabolism, pyruvate metabolism, and amino acid biosynthesis, etc. The proteins include ALDOA, LDHA, PGK1, PKM, TPI1 are a key note in the PPI network. HIF-1 pathway is also shown in PPI network.

By using the ClueGO for functional enrichment, the DEPs were mainly related to the pathway of aerobic glycolysis, the release of mitochondrial cytochrome C, and RAC1/PAK1/MMP2 pathway (Figures 3E,F; Supplementary Figure S1). Aerobic glycolysis contains the above metabolic pathways such as glycolysis, gluconeogenesis, pyruvate metabolism, and HIF-1 related pathways (Supplementary Figure S1). The results showed that NaAsO_2_ treatment caused significant changes in metabolism-related pathways in L-02 cells.

### Protein Expression Profiles of DIP + As-Treated Group Compared With As-Treated Group (DIP + As/As Group)

**FIGURE 4:**
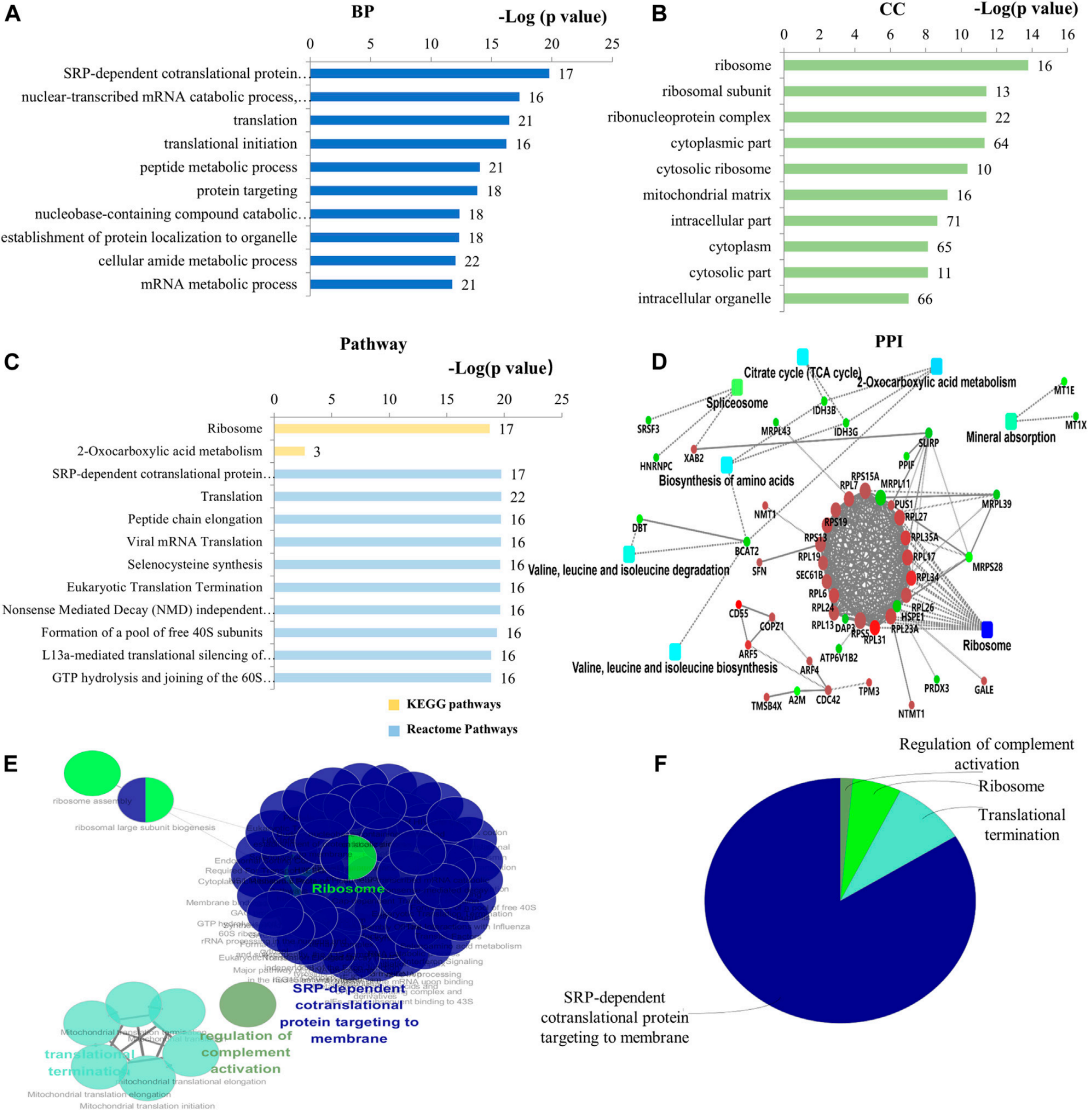
Bioinformatics analysis of the deferentially expressed proteins in DIP + As/As group. **(A)** The top 10 ranking of BP related to the deferentially expressed proteins. **(B)** The top 10 ranking of CC related to the deferentially expressed proteins. **(C)** The significantly enriched pathways associated with the deferentially expressed proteins. KEGG pathway and the top 10 ranking of Reactome pathways. **(D)** PPI network linked to the differentially expressed proteins in DIP + As/As group. Red: the expression of these proteins was up-regulated. Green: the expression of these proteins was down-regulated. **(E)** Functional interaction network analysis was performed by using ClueGO cytoscape plugin. **(F)** Overview the specific cluster. **(A–C)** Number of proteins associated with each category for gene-enrichment analysis is shown on the right of each term bar.

Seventy-one (71) DEPs were identified in the DIP + As/As group. By GO analysis, BP-associated DEPs involve primarily SRP-dependent co-translational protein targeting and membrane, translation, cytoplasmic translation, and mitochondrial gene expression (Figure 4A; Supplementary Table S6). They were widely distributed in cells (Figure 4B,C; Supplementary Table S7) and MF is presented in Supplementary Table S8. Of note, combined GO analysis with UniProt annotation, 21 DEPs were ribosomal proteins (RPs), including DAP3, MRPL11, MRPL39, MRPL43, MRPS28, RPL13, RPL17, RPL19, RPL23A, RPL24, RPL26, RPL27, RPL31, RPL6, RPL7, RPL34, RPL35A, RPS13, RPS15A, RPS19, and RPS5. Among them, 5 DEPs were mitochondrial ribosomal proteins, including DAP3, MRPL11, MRPL39, MRPL43, and MRPS28 (Supplementary Table S7). Based on GO analysis and UniProt annotation, the other 16 were classified as cytoplasmic RPs in the present study. KEGG analysis showed the enrichment of ribosome-related protein translation pathways, nucleotides, and protein metabolism pathways (Figure 4C; Supplementary Table S9), which also are consistent with the GO analysis. In the PPI network analysis, a notable feature was that the DEPs associated with ribosomes were enriched. TCA cycle and amino acid-related biosynthesis pathways were also involved (Figure 4D). Similarly, by using ClueGO for functional enrichment analysis, the DEPs in the DIP + As/As group were highly correlated with the ribosomes (Figures 4E,F; Supplementary Figure S2).

### Protein Expression Profiles of DIP + As-Treated Group Compared With Control Group (DIP + As/Ctrl Group) and the Reversed Proteins Between As/Ctrl Group and DIP + As/As Group

Subsequently, we analyzed the protein expression profiles of DIP + As-treated group compared with the control group. Thirteen (13) DEPs were identified in this group. The relatively small number of DEPs suggested that DIP pre-intervention altered the protein expression profile of L-02 cells exposed to As, making it similar to normal growth L-02 cells. In addition, a total of 14 DEPs overlapped between As/Ctrl group and DIP + As/As group. Among these, 9 proteins were up-regulated and 5 proteins were down-regulated in As/Ctrl group, while their expression trend was reversed in DIP + As/As group. They were named as revered proteins (Supplementary Figure S3A).

GO analysis showed that these 14 revered proteins were widely distributed in cells, and KEGG analysis identified that these reversed proteins were not only primarily involved in the regulation of metabolic pathways and other biological processes, including: 2-oxygen carboxylic acid metabolism, amino acid biosynthesis, citric acid cycle (TCA cycle), but also related to the p53 signaling pathway (Supplementary Figure S3B,C; Supplementary Table S10). The PPI network showed that those proteins were correlated with carbon metabolism, amino acid biosynthetic, TCA cycle, 2-oxycarboxylic acid metabolism, cell cycle, and P53 signaling pathways (Supplementary Figure S3D).

### Hub Gene Analysis and Cluster Analysis of the Expression of Differentially Expressed Proteins in the Key Pathways

**FIGURE 5:**
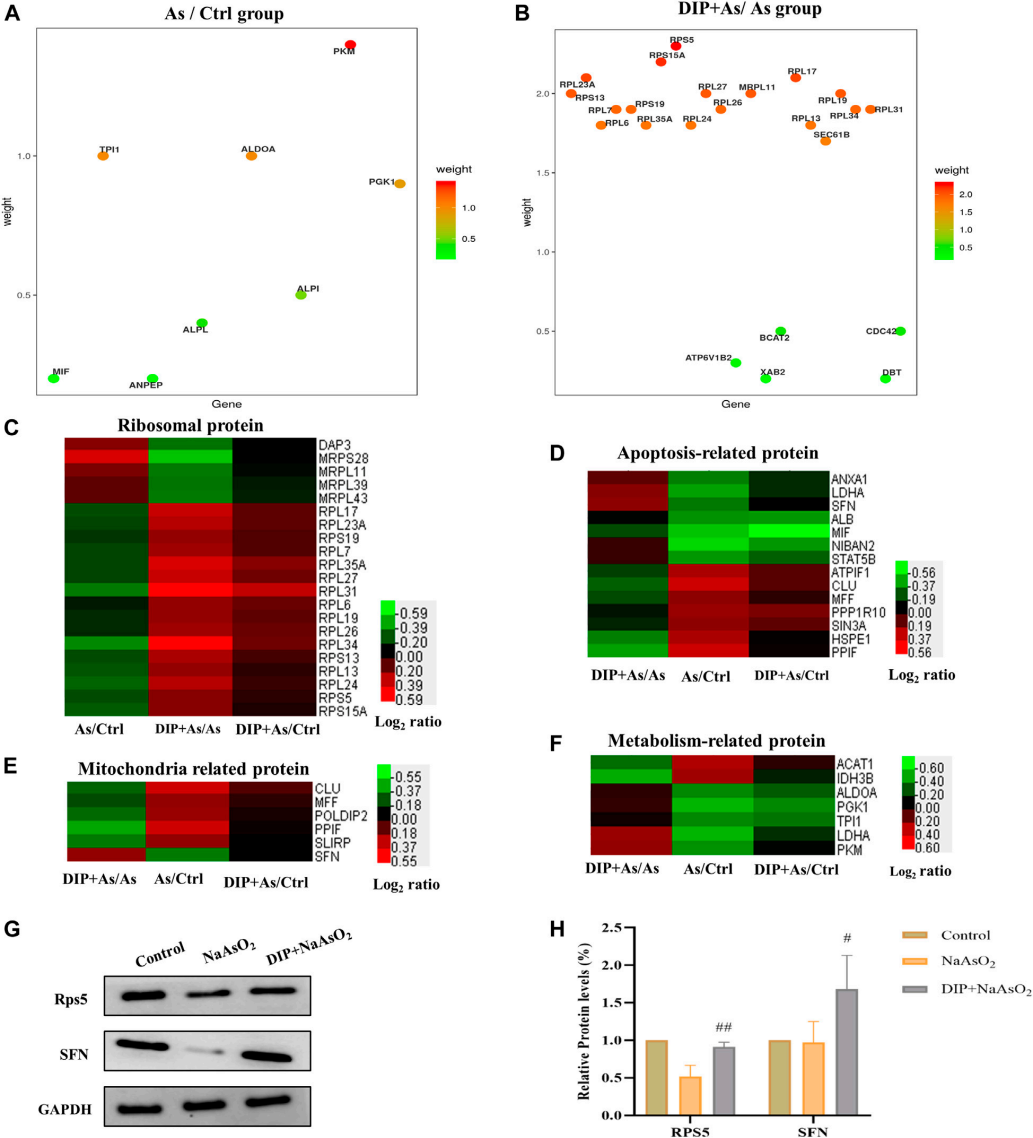
Hub gene and cluster analysis and Western blot analysis verification of DEPs in the key pathways. **(A)** The bubble chart the differentially expressed proteins associated with As/Ctrl group. **(B)** The bubble chart the differentially expressed proteins associated with DIP + As/As group. **(C)** Cluster analysis of ribosomal proteins in different groups. **(D)** Cluster analysis of apoptosis-related proteins in different groups. **(E)** Cluster analysis of mitochondria related proteins in different groups. **(F)** Cluster analysis of metabolism-related protein in different groups. **(G,H)** The differential expression proteins were verified by Western blot analysis.

The hub genes in As/Ctrl group and DIP + As/As group were analyzed. As shown in Figure 5A,B, 8 DEPs (PKM, TPI1, ALDOA, PGK1, ALPI, ALPL, MIF, and ANPEP) were identified as hub genes in As/Ctrl group. Twenty-three (23) DEPs were identified as hub genes in DIP + As/As group. Of these, 16 were ribosomal proteins.

We also analyzed the expression of DEPs in different groups in the key pathways, i.e., ribosomal protein, apoptosis, mitochondria, and metabolism-related protein (Figures 5C–F). The results showed that DIP pretreatment reversed or restored their expression.

### Verification of the Differentially Expressed Proteins by Western Blot Analysis

Based on the above analysis, two hub proteins (RPS5 and SFN), for their largest weight in biological pathways, were selected to be confirmed by Western blot analysis (Figures 5A,B). As shown in Figures 5G,H, consistent with the proteomic results, they were down-regulated in the As/Ctrl group, while DIP pretreatment increased their levels in the DIP + As/As group.

## Discussion

Arsenic is one of the most important toxic elements in the natural environment (Dkhil et al., 2020). So far, endemic arsenism is endangering millions of people worldwide. Arsenic toxicity affects almost all organs, of which the liver is one of the main target organs (Jomova et al., 2011; Samelo et al., 2020). Arsenic toxicity can increase the level of ROS in hepatocytes and cause damage to the mitochondrial respiratory chain, destroy the homeostasis of glucose in the liver, and induce oxidative stress (Rezaei et al., 2019), and result in apoptosis towards the cell caused by ROS (Chirumbolo and Bjorklund, 2017). As expected, here, arsenic exposure induced the apoptosis of L-02 cells. After arsenic treatment, the Bax was increased and Bcl-2 was down-regulated, thus facilitating cytochrome c release. Cytochrome c release increases caspase-3 activation, resulting with DNA fragmentation and chromatin concentration, and ultimately inducing apoptosis (Chimenti et al., 2018; Kalpage et al., 2019). However, DIP inhibited the NaAsO_2_-induced apoptosis in L-02 cells. It antagonized up-regulated Bax expression and down-regulated Bcl-2 expression in L-02 cells exposed to NaAsO_2_. DIP may serve as a potential scavenger for products of oxidative stress (Deng et al., 2012), thereby reducing ROS levels to ameliorate L-02 cell apoptosis caused by arsenic.

Interestingly, by proteomic analysis, 6 DEPs (CLU, MFF, POLDIP2, PPIF, SFN, and SLIRP) in As/Ctrl group were found to be associated with mitochondrial cytochrome c release, apoptotic mitochondrial changes, and mitochondrion morphogenesis, thereby contributing to sodium arsenite induced apoptosis of L-02 cells. In addition, the DEPs related to metabolic pathways were also identified in this group. The pathways mainly included glycolysis/gluconeogenesis, carbon metabolism, pyruvate metabolism, HIF-1 signaling pathway, and biosynthesis of amino acids. These pathways have been observed in a proteomics study of arsenic-induced liver fibrosis in rats (Wu et al., 2018) or yeast (Guerra-Moreno et al., 2015). We found that most of the related proteins were down-regulated, including ALDOA, LDHA, PGK1, PKM, TPI1. This indicates that after NaAsO_2_ treatment, the metabolism-related pathway of L-02 cells decreases. It is consistent with previous study that arsenic inhibits ATP production, particularly through glycolysis or pyruvate metabolism (Guerra-Moreno et al., 2015; Wang et al., 2020b). ALDOA and PKM have been observed to be down-regulated in the above arsenic-treated yeast cells study (Wu et al., 2018). Therefore, in the present study, the effect of arsenic tends to reduce the metabolism of L-02 cells. However, low-level arsenic exposure (75 ppb) can induce aerobic glycolysis (Warburg effect), which is a common phenomenon in cultured human primary cells and cell lines (Zhao et al., 2013). Taken together, these results suggest that when arsenic causes apoptosis, it may cause a decrease in cell metabolism, while at lower concentrations it may cause aerobic glycolysis.

Moreover, 14 DEPs in As/Ctrl group were found to be associated with apoptosis, suggesting that they participate in the process. According to Uniprot protein database (https://www.uniprot.org/), in terms of the function of these proteins, they may play a key role in arsenic-induced L-02 apoptosis. For example, SIN3A is a transcriptional repressor; MFF plays a role in the division of mitochondria and peroxisomes; PPIF is involved in the regulation of mitochondrial permeability transition pore (mPTP); PPP1R10 is a scaffold protein, which plays a role in the control of chromatin structure and cell cycle progression. The expression of these 4 proteins increased in the As/Ctrl group, and they may be related to the apoptosis of L-02 cells induced by NaAsO_2_. On the other hand, the function of STAT5B was signal transduction and transcription activation; NIBAN2 plays a role in the inhibition of apoptosis. Their down-regulation in the As/Ctrl group may also contribute to NaAsO_2_-induced apoptosis. Interestingly, MFF (Li et al., 2019), PPIF (Folda et al., 2016), and STAT5B (Wetzler et al., 2006) have been reported to be associated with arsenic-induced apoptosis.

Of note, 21 DEPs were identified as ribosomal proteins (RPs). Among them, the expression levels of 16 cytoplasmic RPs showed a downward trend. This is similar to two previous studies on the effect of arsenic on yeast cells (Hosiner et al., 2009; Guerra-Moreno et al., 2015), a large number of ribosomal proteins (74 subunits showed a significant reduction) (Guerra-Moreno et al., 2015) or genes (Hosiner et al., 2009) showed a significant reduction after treatment with arsenic. RPs are essential components of the ribosome that comprise a family of RNA-binding proteins involved in modulating a wide variety of biological processes (Ji et al., 2019). This reduction in ribosome abundance may reflect an adaptive response and is a mechanism that protects cells against its toxicity (Guerra-Moreno et al., 2015). On one hand, ribosome biogenesis is a major consumer of cellular energy and RNA polymerase II activity. The down-regulation of the ribosome itself may lead to energy reserves (Gasch et al., 2000) and the expression of other genes as well (Warner, 1999), such as heat-shock proteins (HSPs) (Hosiner et al., 2009). On the other hand, arsenic induces protein misfolding, by reducing the ribosome level, the production of newly synthesized misfolded proteins can be limited, thus allowing the protein degradation pathway to deal with existing misfolded proteins more effectively (Guerra-Moreno et al., 2015). Interestingly, mutants of the ribosome have been observed to be increased arsenic resistant of yeast cell (Dilda et al., 2008; Hosiner et al., 2009; Guerra-Moreno et al., 2015). Other heavy metals such as mercury, nickel (Hosiner et al., 2009), and cadmium (Guerra-Moreno et al., 2015), as well as multiple stress responses or environmental stress (Gasch et al., 2000), can also affect and down-regulate RPs expression. Therefore, ribosome reduction is not specific to arsenic (Guerra-Moreno et al., 2015) and may be a general feature of the environmental stress (Gasch et al., 2000). In addition to cytoplasmic RPs, mitochondria contain ribosomes that synthesize their own proteins. In yeast cells treated with arsenic, most MPRs showed no change in protein abundance (Guerra-Moreno et al., 2015). However, in this study, five MRPs showed an increased expression trend in As/Ctrl group.

Our results indicate that these changes in RPs may be related to the L-02 cell apoptosis induced by arsenic. In fact, apoptosis is particularly sensitive to nucleolar stress signals, and its primary result is the destruction of ribosome synthesis (Rubbi and Milner, 2003). In ribosomal replication disruption, the free ribosomal proteins interact with the p53 system, leading to cell cycle arrest or apoptosis (Warner and McIntosh, 2009). Increasing evidence shows that inhibiting the expression of RPs, such as knocking down many single RPs, will cause p53 accumulation, thereby leading to cell apoptosis (Uechi et al., 2006; Danilova et al., 2008; Daftuar et al., 2013). Interestingly, arsenic has been shown to cause cell death *via* a p53-dependent mechanism (Yu et al., 2008). Among these cytoplasmic RPs, 5 RPs have been reported to be associated with apoptosis, including RPL23 (Dai et al., 2004; Jin et al., 2004), RPL34 (Ji et al., 2019), Ll3a (Chen and Ioannou, 1999), L7 (Chen and Ioannou, 1999), and L35a (Lopez et al., 2002). Inhibition of their expression, such as through siRNA-mediated silencing, leads to cell apoptosis or inhibits cell proliferation (Chen and Ioannou, 1999; Lopez et al., 2002; Dai et al., 2004; Jin et al., 2004; Ji et al., 2019). RNAi-mediating silencing of RPS5 gene expression also resulted in the inability of MEL cells to differentiate (Vizirianakis et al., 2015).

In the view of MRPs, their abnormal expression can cause mitochondrial metabolism disorder and cell dysfunction (Huang et al., 2020). Some MRPs such as MRPS29 (DAP3), MRPL41, MRPS30, and MRPL64 have been identified to be associated with apoptosis by p53 pathway (Yoo et al., 2005; Yan et al., 2017; Huang et al., 2020). In this study, three MRPs (i.e., DAP3, MRPL39, and MRPS28) showed an up-regulated trend in As/Ctrl group. They may be related to L02 cell apoptosis induced by NaAsO_2_. DAP3 (death-associated protein 3), also known as MRPS29; its high expression can promote apoptosis (Miyazaki et al., 2004). Another protein MRPL39 was reported to serve as a tumor suppressor (Yu et al., 2018). Likewise, the inhibition of MRPS28 is related to the treatment of glioblastoma with Benzyl isothiocyanate (BITC) (Tang et al., 2016).

Indeed, the relationship between ribosomes and cell proliferation and apoptosis has been well reviewed (Warner and McIntosh, 2009; Kim et al., 2017; Huang et al., 2020). However, most of the studies on the effects of arsenic on ribosomes involve yeast and tumor cells (Chen and Ioannou, 1999; Gasch et al., 2000; Haugen et al., 2004; Hosiner et al., 2009; Guerra-Moreno et al., 2015; Kim et al., 2017). To the best of our knowledge, the present study is the first to report that arsenic induces a broadly decrease in cytoplasmic RPs, and an increase in mitochondrial RPs in liver cells, resulting in cell apoptosis. Our finding supports this opinion that a number of RPs have secondary roles regardless of their presence in protein biosynthesis, regulation of cell proliferation, or in some cases acting as inducers of cell death (Chen and Ioannou, 1999). Interestingly, the expression of these RPs was reversed in the DIP + As/Ctrl group, suggesting that they may be responsible for DIP attenuating arsenic-induced apoptosis of L-02 cells.

Furthermore, we noted that several proteins may play important roles in arsenic-induced apoptosis and/or DIP resistance to arsenic-induced apoptosis, including ALPI, ALPL CDC42, EIF5A, HSPE1, and SFN. Arsenic sensitivity to enhanced alkaline phosphatase (ALP) activity has been reported (Herrera et al., 2013), where alkaline phosphatase ALPI and ALPL have been shown to be up-regulated in treated cells, whereas DIP pretreatment has reversed its expression. CDC42 is a small GTPase of Rho family, involved in regulation of various functions including positive regulation of cell growth. Here, it was up-regulated in DIP + As/Ctrl group and may be linked to the intervention of DIP on arsenic-induced apoptosis. Two translation initiation factors, eIF2E and eIF4E, have been reported to be related to arsenic-induced cytotoxicity and cell death (Othumpangat et al., 2005; Guerra-Moreno et al., 2015). Here, down-regulation of EIF5A in As/Ctrl may contribute to the NaAsO_2_-induced L-02 cell apoptosis. Arsenic can also cause protein misfolding and induce an increase in HSP protein expression (Tam and Wang, 2020). In this study, the expression of heat shock protein HSPE1 increased in As/Ctrl group. It may participate in endoplasmic reticulum stress and unfolded protein response, and remove misfolded proteins induced by arsenic. SFN (14-3-3 protein Sigma) is one of the isoforms of the 14-3-3 family. This family of proteins regulates a variety of cell functions (Lee et al., 2014). Overexpression of SFN in multiple myeloma cells attenuated arsenic trioxide-induced cell death (Ge et al., 2009). In this study, it was down-regulated in the As/Ctrl group and up-regulated in the DIP + As/Ctrl group, suggesting that it may be involved in NaAsO_2_-induced apoptosis and DIP attenuated apoptosis.

## Conclusion

**FIGURE 6:**
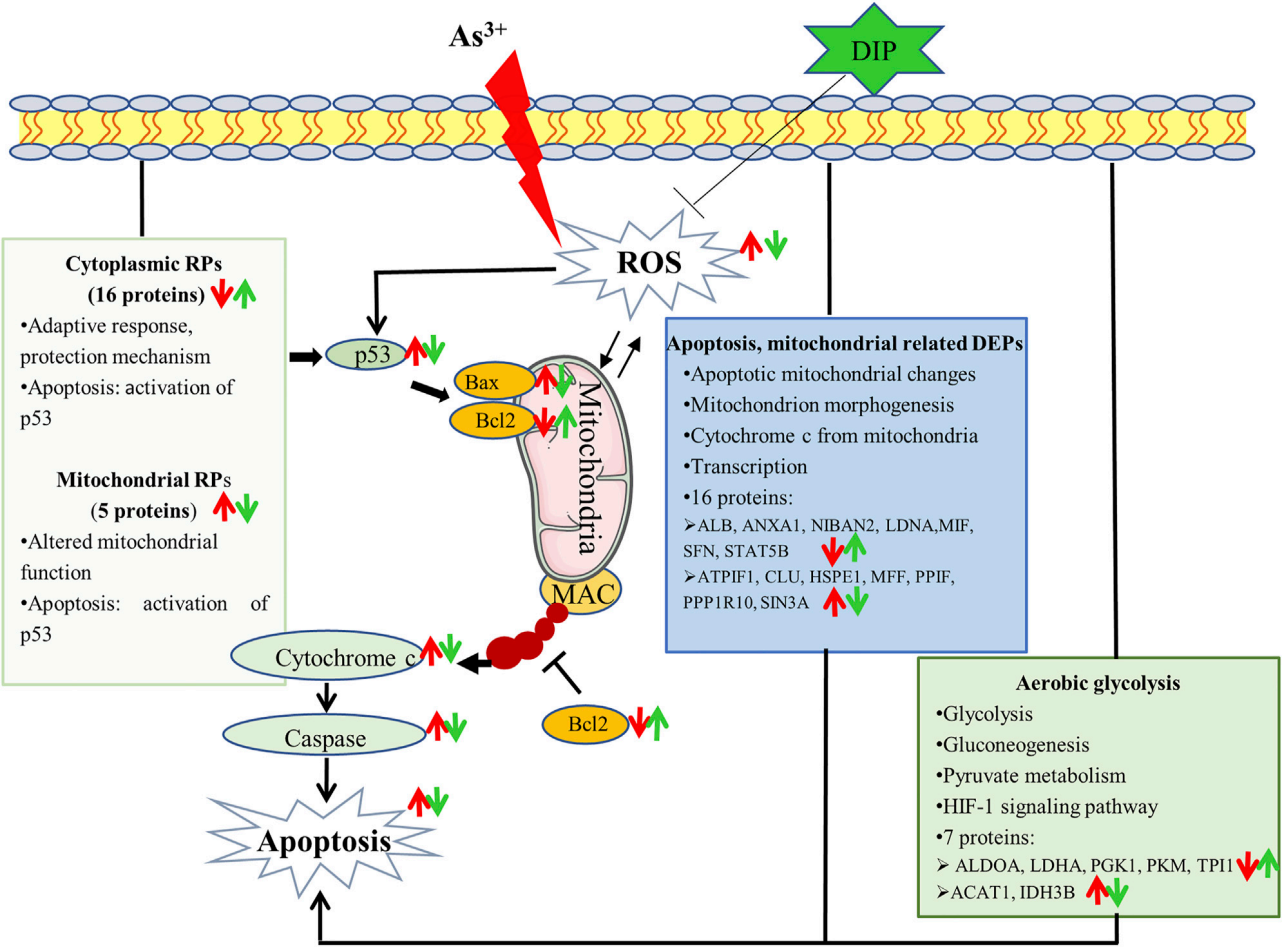
Potential mechanism that sodium arsenite-induced L-02 cell apoptosis and DIP attenuates sodium arsenite-induced apoptosis.: ↑up-regulation.: ↓down-regulation. Red is related to As^3+^, green is related to DIP.

In this study, apoptosis can be induced in human normal liver cells L-02 cells using NaAsO_2_. When hepatic cells were pretreated, the apoptosis was reduced. DIP inhibits cell apoptosis and was associated with an increase in Bcl-2 anti-apoptotic protein and a decrease in Bax pro-apoptotic protein. This may be due to the DIP antioxidant. Proteomic analysis showed that metabolism, apoptosis, and mitochondria-related proteins were associated with arsenic induced apoptosis of L-02 cells. Arsenic concentration induced apoptosis inhibited aerobic glycolysis of L-02 cells. DIP pretreatment reversed or restored the expression of these proteins, suggesting that they were linked to the prevention of DIP apoptosis. Importantly, this is the first study to observe that extensive variations in RPs were associated with arsenic-induced apoptosis in normal human cells. Cytoplasmic ribosomes were down-regulated and mitochondrial ribosomes were up-regulated. The expression of these proteins was reversed by DIP pretreatment. This is also the first study to relate the influence of DIP on apoptosis and its ability to control RP expression. The pathways by which arsenic induces L-02 cell apoptosis and DIP attenuate arsenic-induced apoptosis are summarized in Figure 6. Moreover, there are limitations in this study. It was carried out on a single cell line and needs to be further verified in more cell lines or biological tissues. The mechanism of arsenic-induced apoptosis and DIP’s inhibition of apoptosis disclosed in this study need to be further clarified. We will also explore the quantity (minimum inhibitory concentration) required for DIP to have a protective effect compared to other biological products.

## Data Availability

The original contributions presented in the study are included in the article/Supplementary Material, further inquiries can be directed to the corresponding author.

## Supplementary Material

Supplementary Figure S1 Specific cluster of ClueGO analysis for the As/Ctrl group.

Supplementary Figure S2 Specific cluster of ClueGO analysis for DIP+As/As group.

Supplementary Figure S3 Bioinformatics analysis of the reversed proteins between As/Ctrl, and DIP+As/As group. **(A)** Cluster analysis of the reversed proteins by using Cluster 3.0. **(B)** The GO annotations of the reversed proteins. **(C)** The KEGG pathways associated with the reversed proteins. **(D)** PPI network analysis of the reversed proteins.
