# Supplementary material for: Protective Effect of Dictyophora Polysaccharides on Sodium Arsenite-Induced Hepatotoxicity: A Proteomics Study
Source: Front Pharmacol. 2021 Nov 26;12:749035. doi: 10.3389/fphar.2021.749035 (PMC8660860; doi:10.3389/fphar.2021.749035)
Supplement: Supplementary file 3 [file Table1.DOCX]

**Tabel S1** The monosaccharide content determination of sporocarp polysaccharide of dictyophora

| Composition | Content (mg/L) | Molar ratio |
| --- | --- | --- |
| D-mannose | 15.27 | 1.53:52.30:2.78:1.00:1.78 |
| D-glucose | 523.57 |  |
| D-galactose | 27.80 |  |
| D-xylose | 10.01 |  |
| L-fucose | 17.84 |  |
