# Supplementary figures and images for: Protective Effect of Dictyophora Polysaccharides on Sodium Arsenite-Induced Hepatotoxicity: A Proteomics Study
Source: Front Pharmacol. 2021 Nov 26;12:749035. doi: 10.3389/fphar.2021.749035 (PMC8660860; doi:10.3389/fphar.2021.749035)

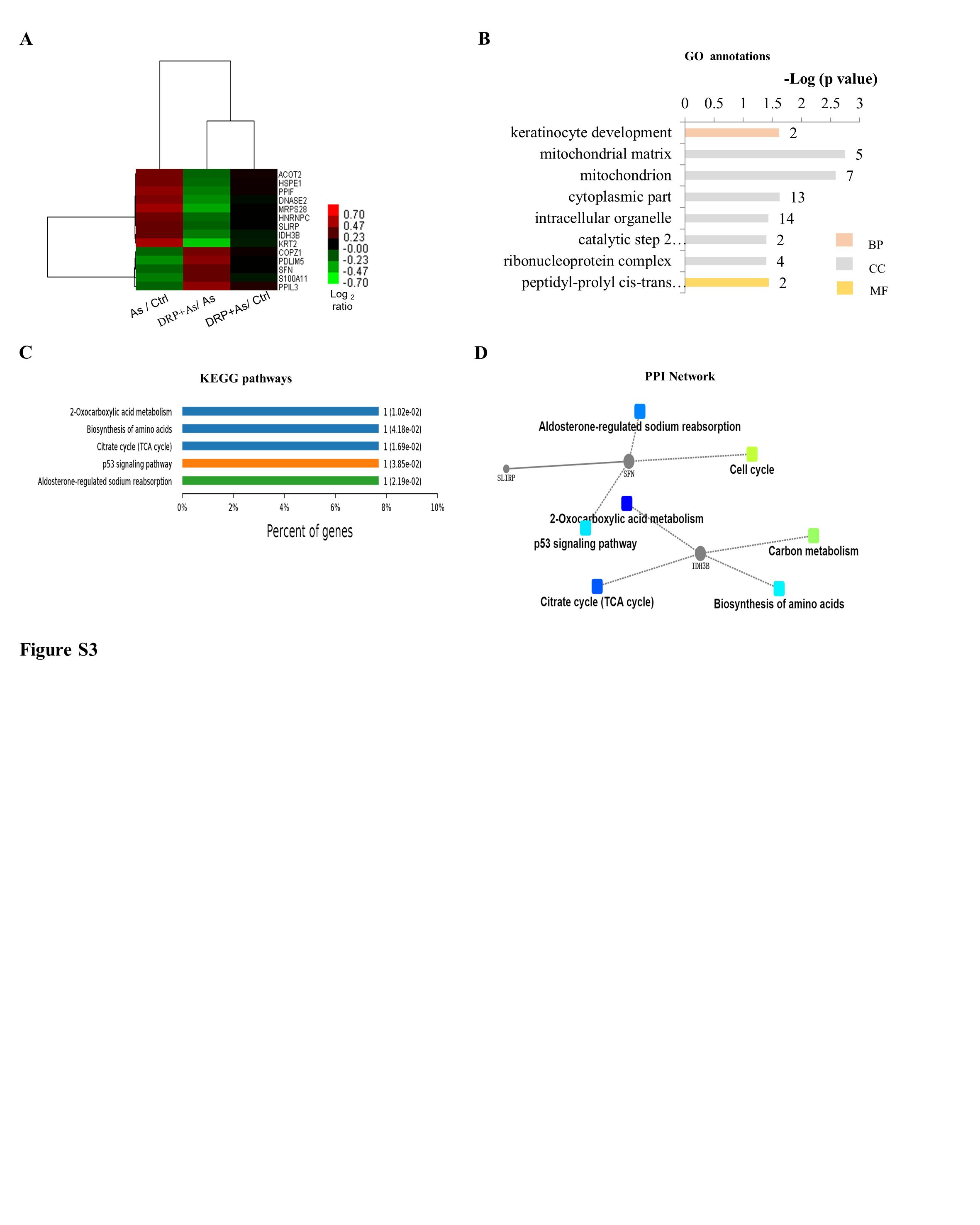

Supplement: Supplementary file 5 [file Image3.TIF]

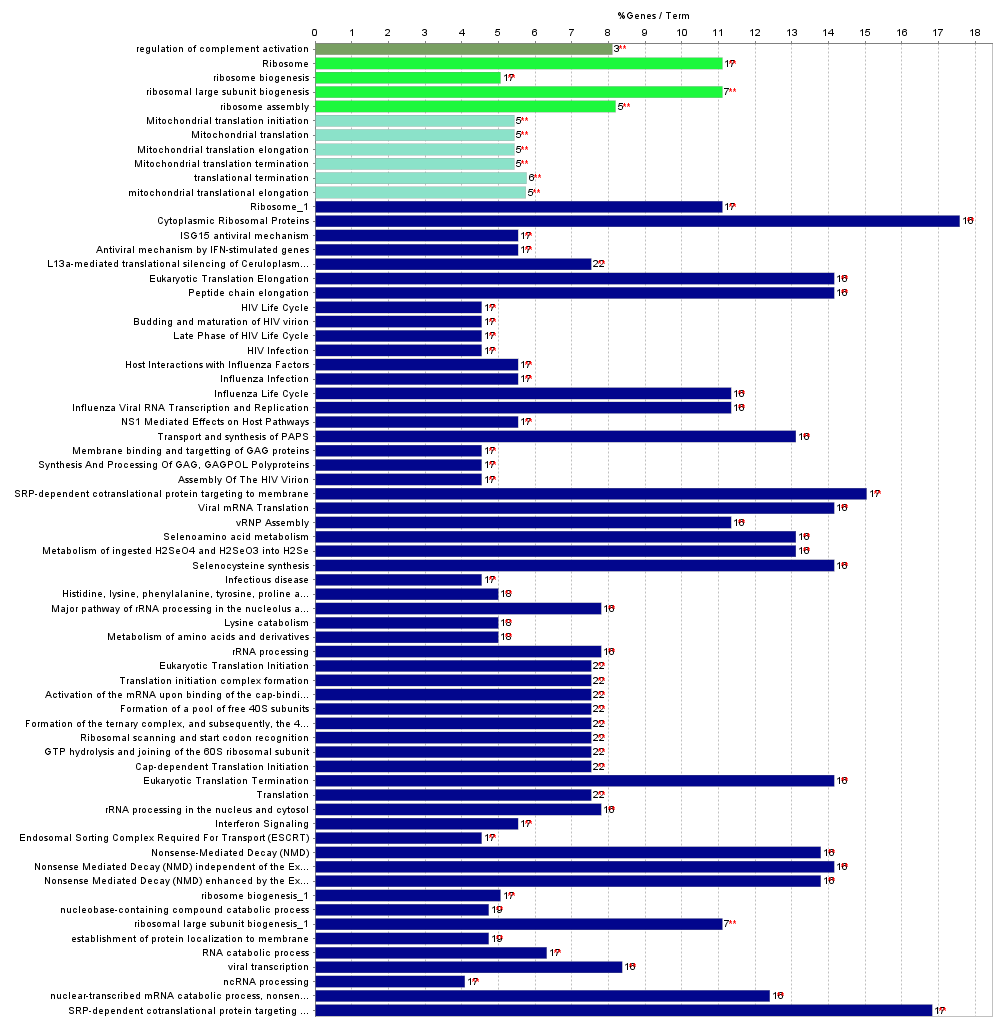

Supplement: Supplementary file 8 [file Image2.PNG]

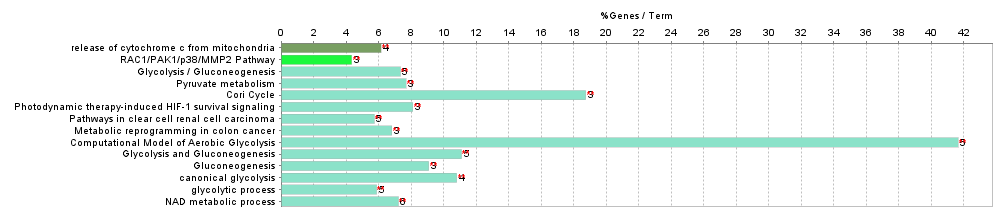

Supplement: Supplementary file 9 [file Image1.PNG]
